# Lower extremity amputation increases oscillatory flow in the infrarenal aorta: A new potential risk factor for abdominal aortic aneurysm development

**DOI:** 10.1186/1532-429X-14-S1-W12

**Published:** 2012-02-01

**Authors:** Alexander V Smolensky, Stephanie Clement-Guinaudeau, Robert W Taylor, John N Oshinski

**Affiliations:** 1 Cardiology, Emory University School of Medicine, Atlanta, GA, USA; 2 Radiology, Emory University School of Medicine, Atlanta, GA, USA

## Background

**Figure 1:**
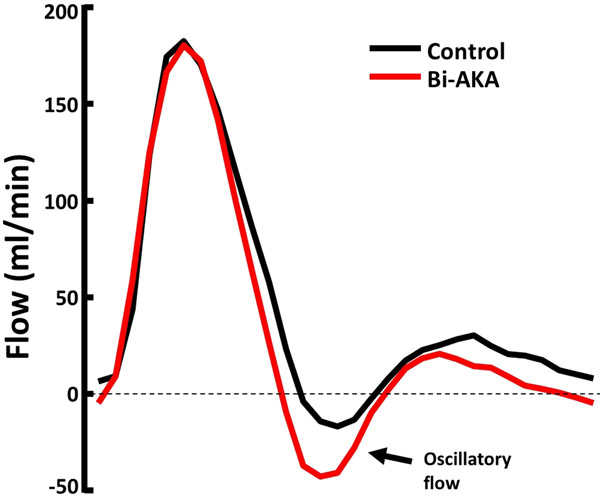


Abdominal aortic aneurysms (AAA) are a major cause of morbidity and mortality in the US. The most common location of AAA is the infrarenal abdominal aorta where oscillatory flow is present over the cardiac cycle, figure 1. Oscillatory flow is known to initiate an inflammatory response in endothelial cells.

Patients with lower extremity traumatic amputations have a 5-fold increase in AAA occurrence unrelated to co-morbidities. The objective of the study is to determine the amount of infrarenal aortic oscillatory flow changes between baseline and acute occlusion of arterial blood supply to a lower extremity (a condition mimicking a lower extremity amputation).

## Methods

Six healthy volunteers underwent MRI exam which included non-contrast MRA and PCMR measurement in the abdominal aorta. PCMR measurements were done at baseline and during 3-4 minutes of acute arterial occlusion using a blood pressure cuff to mimic a unilateral above knee (AKA) and bilateral above knee (Bi-AKA) amputation. PCMR scans were acquired perpendicular to the aorta at 2 levels: below the renal arteries and immediately above the aortic bifurcation. PCMR was used to create waveforms showing flow versus time over the cardiac cycle, figure 1. In all subjects, we computed the percent of retrograde flow over the cardiac cycle at baseline and during the two acute occlusion conditions.

## Results

The retrograde flow was 17.9%+/-1.6 at baseline. AKA resulted in a significant increase in retrograde flow above the aortic bifurcation, the most common site of AAA formation (26.3%+/-4.2 p<0.05). Bi-AKA further increased retrograde flow to 30.1%+/-3.9 p<0.05. Our results suggest that mimicking a traumatic amputation produces a significant increase in infrarenal retrograde aortic blood flow, leading to an increase in oscillatory wall shear stress, a precursor of endothelial dysfunction.

Additionally, the velocity profiles above the aortic bifurcation showed that during unilateral AKA, the diastolic reversal of blood flow was more pronounced on the side of the amputation. These findings agree with clinical observations that amputees are at increased risk for AAA development and that these AAA tend to be asymmetric protruding toward the amputation side.

## Conclusions

Traumatic lower extremity amputation increases retrograde flow in the infrarenal aorta. The mechanism for the increased risk of AAA development in amputees maybe augmentation of retrograde flow in the infrarenal aorta increasing the level of oscillatory wall shear stress.

